# Moving from hand to mouth: echo phonology and the origins of language

**DOI:** 10.3389/fpsyg.2014.00662

**Published:** 2014-07-04

**Authors:** Bencie Woll

**Affiliations:** Deafness, Cognition and Language Research Centre, University College LondonLondon, UK

**Keywords:** sign language, echo phonology, language origins, neuroscience of sign language, mouth gestures

## Abstract

Although the sign languages in use today are full human languages, certain of the features they share with gestures have been suggested to provide information about possible origins of human language. These features include sharing common articulators with gestures, and exhibiting substantial iconicity in comparison to spoken languages. If human proto-language was gestural, the question remains of how a highly iconic manual communication system might have been transformed into a primarily vocal communication system in which the links between symbol and referent are for the most part arbitrary. The hypothesis presented here focuses on a class of signs which exhibit: “echo phonology,” a repertoire of mouth actions which are characterized by “echoing” on the mouth certain of the articulatory actions of the hands. The basic features of echo phonology are introduced, and discussed in relation to various types of data. Echo phonology provides naturalistic examples of a possible mechanism accounting for part of the evolution of language, with evidence both of the transfer of manual actions to oral ones and the conversion of units of an iconic manual communication system into a largely arbitrary vocal communication system.

## Introduction

In the past 50 years, the study of how human language evolved (evolutionary linguistics) has again become a prominent feature of linguistic discourse. A complete theory of language evolution is beyond the scope of this paper, including as it must, consideration of brain function and anatomical changes in the vocal tract. We are concerned here with only one part of the process—the previously hypothesized shift from a primarily gestural or vocal-gestural communication system to spoken language (see section Historical Perspectives below) and how such a shift could have provided a mechanism for converting iconic manual symbols into arbitrary vocal symbols. Data from the sign languages of Deaf^1^ communities will provide an insight into this mechanism.

Since home signing (gesture systems) can appear in the absence of linguistic input (Goldin-Meadow, 2003), sign languages used by Deaf communities have sometimes been regarded as primitive in comparison to spoken languages, and as representing earlier forms of human communication. However, linguistic research over the past 40 years has demonstrated that sign languages are in fact full natural languages with complex grammars (Stokoe, 1960; Klima and Bellugi, 1979; Sutton-Spence and Woll, 1999). The creators and users of all known sign languages are humans with “language-ready brains.” Nevertheless, it is possible that sign share features in common with evolutionary precursors of spoken language.

These features include sharing common articulators with non-linguistic communication (i.e., gestures), and exhibiting substantial iconicity in comparison to spoken languages. This iconicity is present in signs representing abstract concepts as well as in those that represent concrete objects and actions. The form of many signs [examples from British Sign Language (BSL)] depict part or all of a referent or an action associated with a referent, such as eat, paint (holding and using a paintbrush), cat (whiskers), bird (beak). Signs referring to cognitive activities (think, understand, know, learn, etc.) are generally located at the forehead, while signs relating to emotional activities (feel, interested, excited, angry) are located on the chest and abdomen; signs with the index and middle fingers of the hand extended and separated (“V” handshape) relate to concepts of “two-ness”: two, both, two-of-us, walk (legs), look, read (eyes). The pervasiveness of iconicity (even where heavily conventionalized) is striking, in both sign languages and gestures.

If human proto-language was gestural or vocal-gestural, the question remains as to how such a communication system with a high degree of iconicity might link to the development of articulated words in spoken language, in which the links between symbol and referent are, for the most part, seen as arbitrary. Posing the question in this way, and regarding sign languages as “manual” ignores the rich and complex role played by other articulators: body, face, and, in particular, the mouth.

As well as the actions performed by the hands, sign languages also make use of mouth actions of various types. The theory proposed here relates to one subgroup of mouth actions: “echo phonology” (Woll and Sieratzki, 1998; Woll, 2001). These are a set of mouth actions unrelated to spoken language, and which occur obligatorily in a number of sign languages alongside certain manual signs. They are characterized by “echoing” on the mouth certain of the articulatory activities of the hands.

Three data sources are discussed here: narratives in 3 different European sign languages, anecdotal observations of hearing individuals bilingual in BSL and English, and functional imaging studies with deaf signers. These provide evidence of a possible mechanism in the evolution of spoken language by which iconic symbols in a manual communication system could have converted into a vocal communication system with arbitrary links between symbol and referent.

## Historical perspectives

Many writers have suggested that human vocal language may have evolved from manual gestures. What is required to sustain such a claim is a plausible mechanism by which primarily manual actions could have transformed themselves into vocal actions. One mechanism (not even requiring communicative gesturing as an intermediate stage) was suggested by Darwin in *The Expression of Emotions in Man and Animals* (1872):

“there are other actions [of the mouth] which are commonly performed under certain circumstances… and which seem to be due to imitation or some sort of sympathy. Thus, persons cutting anything may be seen to move their jaws simultaneously with the blades of the scissors. Children learning to write often twist about their tongues as their fingers move, in a ridiculous fashion.” (Darwin, 1872, p. 34)

Henry Sweet (1888) extended this notion to encompass a transition from manual gesture to “lingual gesture”:

“Gesture.. helped to develop the power of forming sounds while at the same time helping to lay the foundation of language proper. When men first expressed the idea of “teeth,” “eat,” “bite,” it was by pointing to their teeth. If the interlocutor's back was turned, a cry for attention was necessary which would naturally assume the form of the clearest and most open vowel. A sympathetic lingual gesture would then accompany the hand gesture which later would be dropped as superfluous so that ADA or more emphatically ATA would mean “teeth” or “tooth” and “bite” or “eat,” these different meanings being only gradually differentiated.” (Sweet, 1888, pp. 50–52)

To Sweet, therefore, should go the credit for hypothesizing that a “lingual gesture accompanying a natural hand gesture” could be a key link between gesture and spoken language. However, he provides no evidence for such a process, failing to explain more general features of what he calls sympathetic lingual gestures.

Richard Paget (1930) attempted to find evidence for such a theory. Like Sweet, Paget claimed that the earliest human language was a language of gestures, in which manual actions were unconsciously copied by movements -of the mouth, tongue, or lips.

“Originally man expressed his ideas by gesture, but as he gesticulated with his hands, his tongue, lips and jaw unconsciously followed suit… The consequence was that when, owing to pressure of other business, the principal actors (the hands) retired from the stage… their understudies—the tongue, lips, and jaw—were already proficient in the pantomimic art.” (Paget, 1930, p. 133)

He supplies a number of examples of this process:

“Another … example may be given, namely, in connection with the beckoning gesture—commonly made by extending the hand, palm up, drawing it inwards toward the face and at the same time bending the fingers inwards toward the palm. This gesture may be imitated with the tongue, by protruding, withdrawing, and bending up its tip as it re-enters the mouth.

If this “gesture” be blown or voiced, we get a resultant whispered or phonated *word*, like **edə**, **eđə**, or **eđra** … suggestive of … our English word “hither”.” (Paget, 1930, p. 138)

Paget's theory (known as the “ta-ta” theory from another example suggesting parallels between waving goodbye and flapping the tongue) was developed further by Swadesh (1971). He provides another example of its application:

“… a word like the Latin *capio*, I take, or English *capture*, whose root begins with a *k* sound and ends in the sound *p*, made by closing the lips. It has been suggested that the formation of the *k* sound at the back of the mouth, while the lips are open, is comparable to the open hand. The closing of the lips, then, is analogous to the fingers closing with the thumb as one takes hold of an object. Thus the pronunciation of the root *capio* is like the action of taking. Of course not all words are to be explained in this way; in fact, only a few. And yet the possibility that some words developed in this way is not denied by other qualities also evident in language.” (Swadesh, 1971, p. 4)

Paget's theory can only be validated if there is evidence for a historical process by which manual gestures were reflected in movements of the lips and tongue, which were in turn associated with the production of speech-sounds. One weakness of the approach of Paget and the others is that they all suggest that the mouth actions share underlying imagery with the associated iconically-motivated manual gesture, leaving open the question of how a hypothesized highly iconic manual communication system could have subsequently led to spoken language, with its generally arbitrary links between symbol and referent.

Hewes (1973) serves as a point of connection between the writers of the late nineteenth and early twentieth century and contemporary writings on language evolution. Kendon (2010) in a review of Fitch (2010) summarizes Hewes' view that primate gestures served as a better point of comparison with human language than their vocalizations. Hewes did recognize, however that a challenge for a gestural origin of human language was the need to account for the switch from manual to vocal communication. His suggested reasons included the greater convenience of speaking (it could be used in the dark and while the hands were occupied), and an increase in vocabulary and ease of lexical retrieval. He also supported Paget's (1930) hypothesis, discussed above.

Recent studies (Erhard et al., 1996; Rizzolatti and Arbib, 1998; Rizzolatti and Craighero, 2004) provide such evidence of links between brain areas associated with language and areas controlling movement of the hands and arms (also see below). However, such findings have not been used to suggest a mechanism in language evolution for the twin shifts from hand to mouth and from iconic to arbitrary symbols.

## Contemporary evidence

### Neurobiological perspectives

Studies of neurons in the monkey brain by Rizzolatti and colleagues since 1996 (Rizzolatti et al., 1996; Rizzolatti and Craighero, 2004) have identified “mirror neurons,” which fire when a primate observes another individual (monkeys and humans) making specific reaching and grasping movements. The mirror system, in temporal, parietal, and frontal regions, is part of a system specialized for perceiving and understanding biological motion. Although research has not shown a mapping of vocalization production onto perception of vocalizations, this mapping is implicit in Liberman and Mattingly's (1985) motor theory of speech perception, which proposes that speech is understood in terms of its articulation, rather than its perception. It should also be noted that the anatomical proximity of neurons in the premotor cortex relating to hand and mouth functions may relate evolutionarily to the involvement of both in activities such as eating. The relationships between mouth actions related to eating, and mouth actions found in spoken language, have been discussed in detail by MacNeilage (1998). Meguerditchian and Vauclair (2008), describe shared features in the co-occurrence of manual and vocal gestures in non-human primates.

In a series of studies, Gentilucci and colleagues have shown that mouth actions are related to manual actions. When participants were asked to grasp objects of different sizes while articulating syllables such as /ba/ there was a parallel increase in the mouth opening and voice spectra of syllables pronounced simultaneously. Semantically congruent words and gestures also show interaction effects not seen in incongruent pairings (Gentilucci, 2003; Gentilucci and Corballis, 2006; Barbieri et al., 2009). Bernardis and Gentilucci (2006), describing the relationship of words and emblems in processing and execution, hypothesize that a system relating actions to syllables might have evolved into a system relating symbolic gestures to words, and importantly, draw on neurological evidence about the role of Broca's area in both gesture and language.

### Gesture and speech

A number of theorists have postulated that manual gesture (on its own, without consideration of vocalization or mouth gesture) is the origin of language. Rizzolatti and Arbib (1998) align with the earlier nineteenth and twentieth century writers, seeing gesture as fading once speech has emerged:

“Manual gestures progressively lost their importance, whereas, by contrast, vocalization acquired autonomy, until the relation between gestural and vocal communication inverted and gesture became purely an accessory factor to sound communication” (Rizzolatti and Arbib, 1998, p. 193).

In such models, gesture is seen as unintegrated with speech—both in modern human communication and in human evolution.

McNeill et al. (2008) provides a strong set of arguments against this position. They argue that a unimodal communication system, using gesture or sign alone, could not have evolved into modern human communication, which is primarily bimodal (gesture and speech). They suggest that if such a phase existed, it was not a proto-language, but a precursor of mimicry and pantomime. They argue that a “gesture-first” theory:

“incorrectly predicts that speech would have supplanted gesture, and fails to predict that speech and gesture became a single system. It is thus a hypothesis about the origin of language that almost uniquely meets Popper's requirement of falsifiability—and *is* falsified, doubly so in fact. (McNeill et al., 2008, p. 12)”

Another thread in the “supplantation of gesture by speech” argument relates to the advantages of speech over gesture (Corballis, 2003). McNeill et al. (2008) have argued that speech is the default form of human communication because it has fewer dimensions, is more linear, is non-imagistic (and hence more arbitrary, with the potential for a larger lexicon), etc. Given this asymmetry, McNeill and colleagues argue that even though speech and gesture are selected jointly, it would still be the case that speech is the medium of linguistic segmentation:

“Sign languages—their existence as full linguistic systems—impresses many as a reason for gesture-first, but in fact, historically and over the world, manual languages are found only when speech is unavailable; the discrete semiotic then transferring to the hands. As we shall see later, this transfer takes place automatically. So it is not that gesture is incapable of carrying a linguistic semiotic, it is that speech (to visually disposed creatures) does not carry the imagery semiotic.” (McNeill et al., 2008, p. 13)

## Hands and mouth in sign language

### Mouth actions and other non-manual articulators

**Figure 1:**
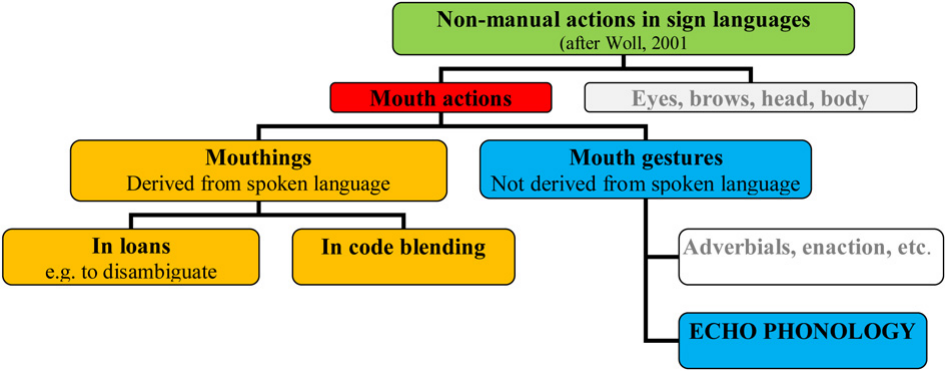
**Mouth actions in sign language**.

As mentioned above, sign languages of the deaf offer a unique perspective on language, since they embody the structural and communicative properties of spoken language, while existing entirely within a wholly visual-gestural medium. Among other insights, they enable investigators to clarify the core components of language in distinction to those that reflect input or action characteristics of the language system. This difference is reflected in the articulators on which languages in the two modalities rely. Sign languages use both manual and non-manual articulators, including the head, face and body (e.g., Liddell, 1978; Sutton-Spence and Woll, 1999). Within the face, eye actions such as eye narrowing, changes in direction of gaze and eyebrow actions (raise/lower) play important roles in SL communication (Crasborn, 2006). In addition, although sign languages are not historically related to the spoken languages of their surrounding hearing communities, sign languages do borrow elements from spoken language (Sutton-Spence and Woll, 1999). Thus some mouth actions (mouthings) are derived from spoken language, while other mouth actions (mouth gestures) are unrelated to spoken languages (see Figure 1 below).

In a study of narratives in three European sign languages (Crasborn et al., 2008) mouth actions were found throughout (Table 1). There is striking uniformity in the percentage of signs accompanied by mouth gestures (35–39%), with greater variation across the three languages in the percentage of signs accompanied by mouthings (26–51%).

**Table 1:** **Comparison of hand/mouth actions in three sign languages**.

**Language**	**Number of Manual signs**	**Number of mouth gestures**	**% of signs accompanied by mouth gestures**	**Number of mouthings**	**% of signs accompanied by mouthings**
British Sign Language	1552	539	35	560	36
Sign Language of the Netherlands	1162	458	39	299	26
Swedish Sign Language	1619	624	39	831	51

#### Mouthings

Sign languages can borrow mouth actions from spoken words—speech-like actions accompanying manual signs that can disambiguate manually homonymous forms. These are considered to be borrowings, rather than contact forms reflecting bilingualism in a spoken and signed language, since there is evidence that signers can learn these without knowing the source spoken language. These serve to disambiguate “manual homonyms”: signs with similar or identical manual forms. For example, the BSL signs asian and blue, are manually identical (see **Figure 3C** below). To distinguish which meaning is meant, mouthings are incorporated, derived from the mouth actions used when speaking the words “Asian” or “blue.”

#### Adverbials

Adverbials are arrangements of the mouth which are used to signal manner and degree (e.g., to indicate that an action is performed with difficulty or with ease; to indicate if an object is very small or very large, etc.). In **Enaction** (sometimes called mouth-for-mouth), the action performed by the mouth represents that action directly (e.g., in chew, the mouth performs a “chewing” action, while the sign is articulated on the hands).

## Echo phonology

The term **Echo Phonology** (Woll and Sieratzki, 1998; Woll, 2001, 2009) is used for a class of mouth actions that are obligatory in the citation forms of lexical signs. In the BSL sign true (see **Figure 3D** below), the upper hand moves downwards to contact the lower hand, and this action is accompanied by mouth closure, synchronized with the hand contact. This category of mouth gesture differs from adverbial mouth arrangements as the mouth gesture forms part of the citation form of the manual sign, and unlike adverbial mouth gestures, do not carry additional meaning. Crasborn et al. (2008) refer to this category of mouth gestures as “semantically empty.” Signs with echo phonology appear incomplete or ill-formed in their citation form if the mouth gesture is not present.

The term “echo phonology” is used, since the mouth action is a visual and motoric “echo” of the hand action in a number of respects: onset and offset, dynamic characteristics (speed and acceleration) and type of movement (e.g., opening or closing of the hand, wiggling of the fingers). Echo phonology mouth gestures are not derived from or related in any other way to mouth actions representing spoken words; in the citation form of these signs they are an obligatory component, and are presumably constrained by the common motor control mechanisms for hands and mouth discussed above. The citation forms of the signs in which they are found require the presence of the mouth gesture to be well-formed, and the mouth gesture always includes some movement such as inhalation or exhalation, or a change in mouth configuration (opening or closing) during the articulation of the sign: for example, BSL signs exist (wiggling of fingers, no path movement, accompanied by [⨜]); true (active hand makes abrupt contact with palm of passive hand, accompanied by [am]—see **Figure 3D** below); disappear (spread hands close to “flat o” shape, accompanied by [θp]).

The essential dependence of the mouth gesture on the articulatory features of the manual movement can be seen in three BSL signs all meaning “succeed” or “win.” Three different oral patterns of mouthing co-occur with these signs, and one cannot be substituted for the other. In succeed, the thumbs are initially in contact, but move apart abruptly as the mouth articulates [pa]. In win, the hand rotates at the wrist repeatedly as the mouth articulates [hy]; and in won, the hand closes to a flat o, while the mouth articulates [∧p]. Most importantly, the action of the mouth in signs with echo phonology, while echoing that of the hands, is not in itself iconic.

**Figure 2:**
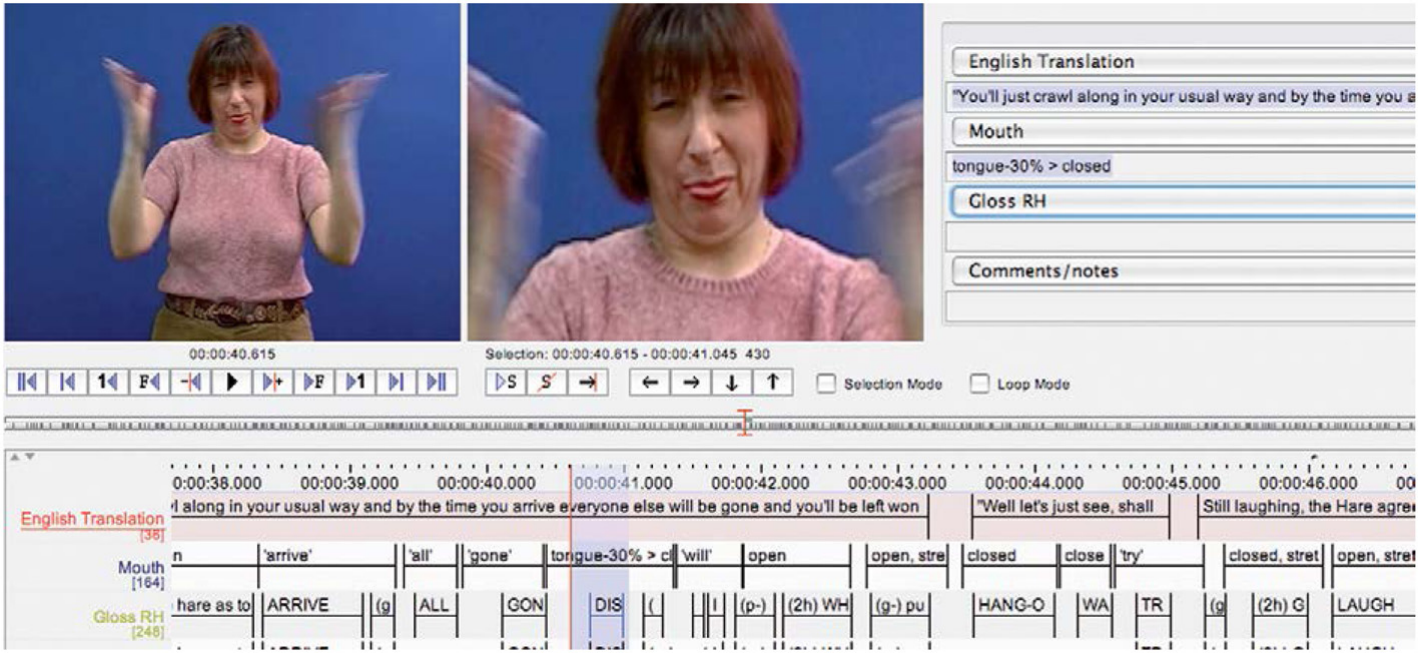
**BSL sign disappear showing initial configuration of mouth**.

The parallel movements of the hands and mouth found in echo phonology can also be seen in the production of the BSL sign disappear (Figure 2). Both hands are open and the tongue is protruding at the onset of the manual sign. The notation tiers show that during the movement of the sign, as the hands close, the tongue retracts.

### Syllables occurring in echo phonology in BSL

The following elements (Table 2) have been identified, although it is likely that this is not an exhaustive list. It is not known what inventories exist in other sign languages. Some articulatory features are given for them; and since echo phonology is a feature of a language used by deaf people, no voiced-voiceless distinction is operative and almost all involve articulations at the front of the mouth or lips, where they are most visible.

**Table 2:** **Echo phonology elements in BSL**.

**CONSONANTS—SYLLABLE-INITIAL**	
p	Bilabial stop
f	Labio-dental stop
**CONSONANTS—SYLLABLE-FINAL**	
p	Bilabial stop
m	Bilabial nasal
ς	Glottal stop
**VOCALICS**	
y	Front rounded vowel
∧ or a	Low central vowel
u	Back rounded vowel
h	Pharyngeal fricative
w	Bilabial fricative
θ	Interdental fricative
⨜	Rounded palatal fricative
**BREATH PATTERNS**	
<	Exhalation
>	Inhalation

The combinations of these elements result in syllables. Selected examples of signs using these syllables are given (Table 3).

**Table 3:** **Examples of syllables with echo phonology**.

<	done
pa	
Associated with one or two active hands, movement consists of hand separation and twisting, with single sharp action	
< < <	not-bothered
fu *or* fw *or* fy	
<	exist
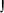	
Wriggling or fingers, repeated shaking or twisting of wrists(s), no path movement	
< <	win
hw *or* hy	
Repeated twisting of wrist, no path movement	
>	thank-god
∧p	
Closing and twisting of hand(s), sharp movement	
> >	true
am *or* ∧m	
Hand closes and contacts passive hand, sharp movement	
>	disappear (also see Figure 2)
θp	
Hand(s) close, sharp movement with abrupt stop	

Although echo phonology is largely voiceless in deaf signers, hearing people with deaf parents (bilinguals native in both BSL and English) frequently mix sign and speech, either in the form of code-mixing (switching between English and BSL) or—because these languages occur in different modalities (bimodal bilingualism)—by means of code blending, where elements from a spoken language appear simultaneously with elements of a sign language.

Anecdotal observations from conversations between hearing people with deaf parents (bilinguals native in both BSL and English) indicate that echo phonology appears (with or without voicing) in the form of code mixing with English in the absence of production of the manual component. In other words, only the oral component is produced.

Examples include:

1. A: “Have you done that poster?”
  B: “[⨜⨜⨜] (NOT-YET), I'll do it tomorrow” (voiceless)
2. A: “It was terrible. [**∧**mp]”' (END/absolutely over) (voiced)
3. A: “I couldn't get a straight answer from anyone. It was completely [pıpıpı]” (VARIED/inconsistent) (voiced)

These examples are suggestive of a possible leap from echo phonology in signs to a situation where voicing accompanies these mouth gestures so that they begin to have independent existence as lexical items. Further research is necessary to explore whether these forms are more similar to vocal gestures or to words.

Sweet, Paget and the other early writers cited above postulated that iconicity in the mouth gesture itself was the source of spoken words. However, it is difficult to see how a mouth gesture on its own could iconically express the semantic notion of “succeed” or “true.” Echo phonology illustrates a mechanism by which abstract concepts, which can be represented by iconic manual gestures, can be attached to abstract mouth gestures.

## Echo phonology in different sign languages

In a study comparing narratives in three sign languages, the occurrence of echo phonology was compared with other types of mouth action. The data are drawn from the ECHO (European Cultural Heritage Online) corpus. This corpus was created as part of a European Union pilot project with the aim of demonstrating how scientific data within the humanities (including linguistics) can be made widely accessible via the Internet (Crasborn et al., 2007).

Data were collected from one male and one female Deaf native signer of each of BSL, NGT, and SSL—a total of six signers. After reading brief summaries in order to familiarize themselves with the content, signers were asked to sign to camera their own versions of five of Aesop's fables. Data were then coded with ELAN software, using a broadly defined set of transcription categories. In all, 51 min of signed material were included in this study. All annotated data from this study is freely available at the ECHO web site: http://www.let.ru.nl/sign-lang/echo.2

Echo phonology was found in all three sign languages. Of mouth gestures found in the narratives (i.e., excluding signs with mouthing), signs with echo phonology form 10.8% of mouth gestures in BSL, 12.6% in Sign Language of the Netherlands, and 16% in Swedish Sign Language (Crasborn et al., 2008).

Echo phonology has also been studied in other sign languages, including German Sign Language (Pendzich, 2013) and American Sign Language (Mather and Malkowski, 2013). Mather and Malkowski explored opening and closing movements of the mouth in detail, in particular, how mouth closing occurs when the hands contact the body, and mouth opening occurs when hand contact with the body is broken.

## Neural correlates of echo phonology

Despite the differences in the modality of the perceived signal, the neural organization of language is remarkably similar in spoken and signed language. Neuroimaging studies of native signers show similar patterns of lateralization and activation when processing spoken or signed language data. Specifically, sign language processing is associated with activation in left temporal and frontal cortex, including Broca's area (BA 44/45), just as for spoken language (see e.g., Emmorey, 2001; Corina et al., 2003; MacSweeney et al., 2008; Newman et al., 2010 for a review). MacSweeney et al. (2002) also found no differences between BSL and English in the extent of lateralization, with both languages left-lateralized. Studies of patients with brain lesions following CVA consistently indicate that perisylvian regions of the left hemisphere support language processing (Atkinson et al., 2005; see Woll, 2012 for a review).

Despite their similarities, the networks for spoken and sign language are not completely identical. MacSweeney et al. (2002) report that regions which showed more activation for BSL than audiovisual English included the middle occipital gyri, bilaterally, and the left inferior parietal lobule (BA 40). In contrast, audio-visual English sentences elicited greater activation in superior temporal regions than BSL sentences (pp. 1589–1590).

With these considerations in mind, Capek et al. (2008) explored the sensitivity of the cortical circuits used for language processing to the specific articulators used, not only comparing speech and signing but examining activation during perception of signs with English mouthing, with echo phonology, and with no mouth actions. In their fMRI experiment, lists of lexical items were presented to deaf native signers. These comprised: (1) silently articulated English words with no hand action (SR); (2) BSL signs with no mouth action (hands only—Man); (3) BSL signs with mouthings (disambiguating mouth, where the mouthing distinguished between two manually identical signs—DM); and (4) BSL signs with echo phonology (EP).

The stimuli were designed to vary on the dimensions of presence or absence of mouth opening/ closing; presence or absence of hand and arm movements; and presence or absence of English-based mouth actions (Table 4).

**Table 4:** **Characteristics of stimuli in fMRI experiment**.

	**Mouth opening and closing**	**Hand- arm movements (BSL)**	**English-derived mouth**
No mouth (HO)	−	+	_
Phonology (EP)	+	+	−
Disambiguating mouth (DM)	+	+	+
Silent speech (SS)	+	_	+

Stimuli consisted of single words/signs, examples of which are given in Table 5. The list of silently spoken words was based on English translations of the signs.

**Table 5:** **Examples of stimuli in fMRI study (EP syllables in brackets)**.

**EP**	**DM**	**Man**
EXIST[ 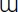 ]	FINLAND/METAL	TABLE
WIN [hy]	BATTERY/AUNT	CHERRY
NONE [pu]	WOOD/PROBLEM	BUTTER
SUCCESS [pa]	RUSSIA/BOY	KNOW
END [pəm]	ITALY/WIN	FAX

**Figure 3:**
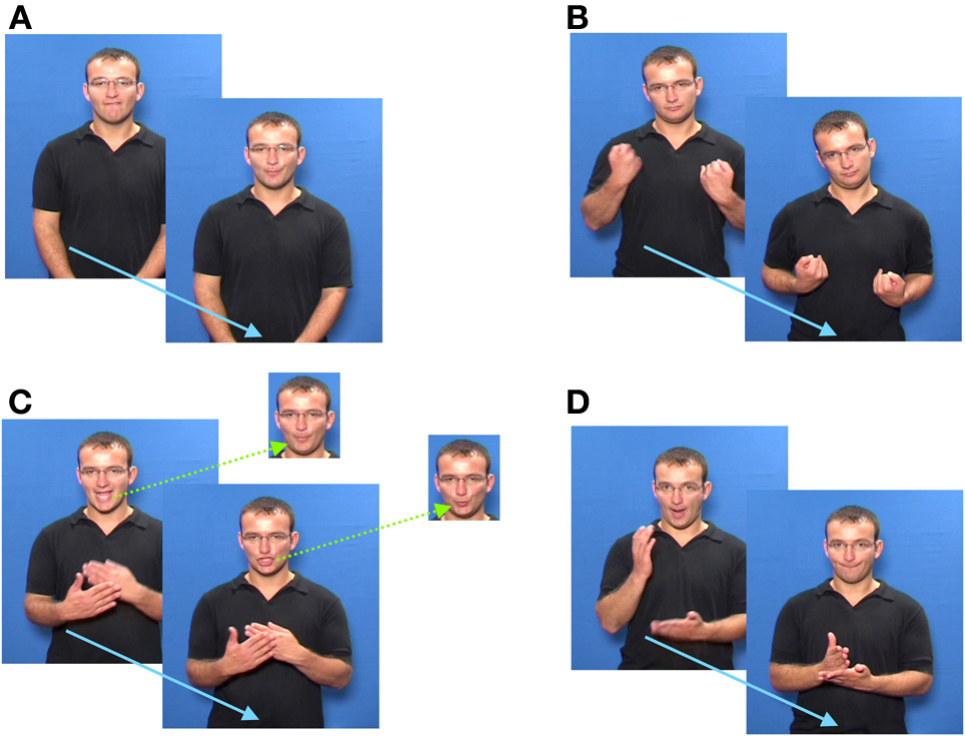
**Illustrations of stimuli**. **(A)** SS, Silent articulation of the English word “football.” The fricative (/f/)(“foot..”), and the semi-open vowel /ɔ:/ (“..ball”) are clearly visible. **(B)** Man, The BSL sign ILL. **(C)** DM, The BSL sign ASIAN shows the mouthing of /eI/ and /ʒ/. The face insets show the corresponding parts of the mouthings for the manual homonym BLUE, where /b/ and /u:/ can be seen. **(D)** EP, The manual sequence for [TRUE] requires abrupt movement from an open to a closed contact gesture. As this occurs, the mouth closes abruptly.

Figure 3 shows examples of each of the stimulus types:

Thirteen (6 female; mean age 27.4; age range: 18–49) right handed participants participated. Volunteers were congenitally deaf native signers, having acquired BSL from their deaf parents. Stimuli were presented in alternating blocks of each of the experimental and a baseline condition. In order to encourage lexical processing, participants performed a target-detection task. Full details of the experimental protocol and analysis may be found in Capek et al. (2008).

### Sign language (Man, DM, EP)

**Figure 4:**
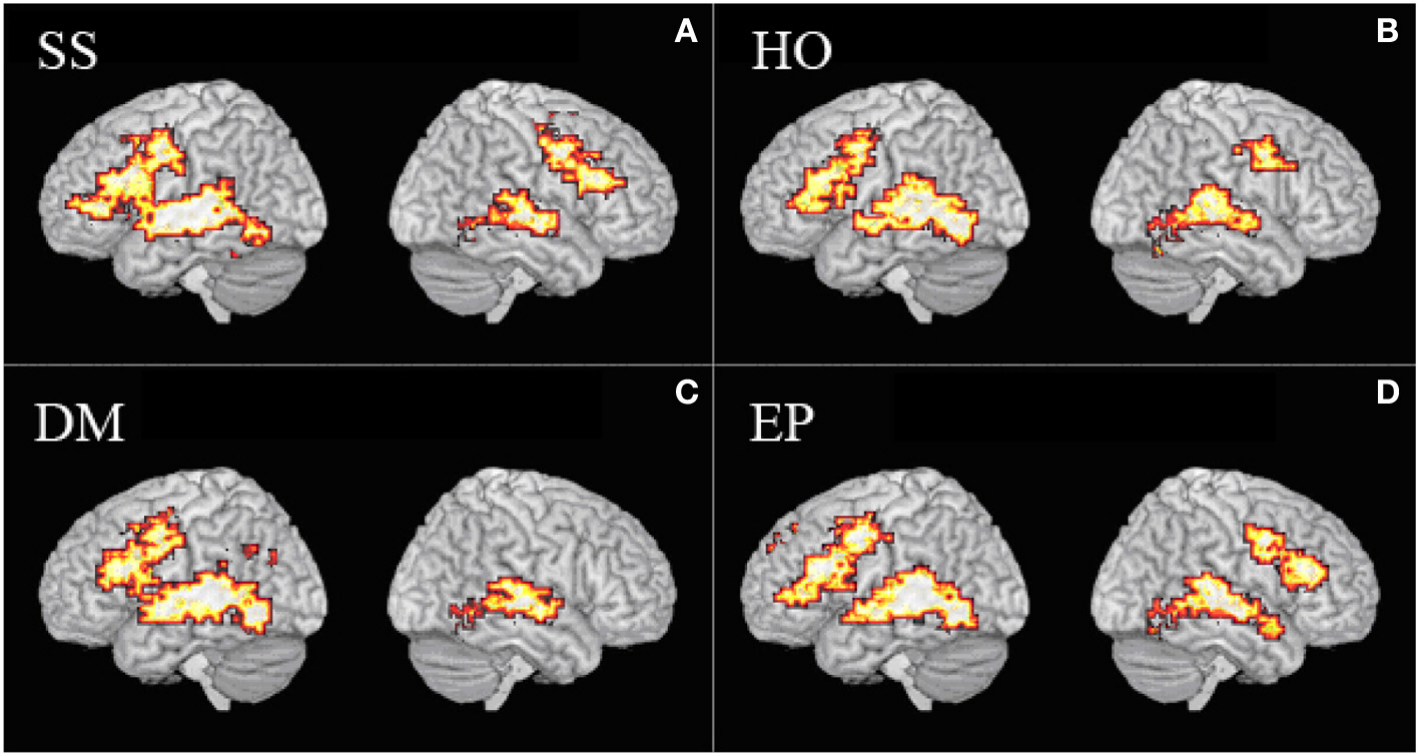
**Brain activation**. **(A)** Activation during silent speechreading (SS). **(B)** Activation during processing of signs without any mouth actions (HO). **(C)** Activation during processing of signs with disambiguating mouth actions (DM). **(D)** Activation during processing of signs with echo phonology (EP).

In all three sign language conditions, Deaf native signers activated core language regions that are typically found when hearing people listen to speech. Although both sign language and speech involve perisylvian regions, sign language perception activated more posterior and inferior regions (Figure 4).

### Comparing echo phonology (EP) and other mouthings (DM)

The task required participants to process material linguistically. In order to achieve lexical processing, BSL users must integrate perceptual processing of hands and of face/head, and this needs to be achieved fluently and automatically. If the cortical circuitry for sign language processing were driven by a mechanism that is “articulation-blind,” we would expect there to be no systematic differential activation between signs with mouthings (where the mouth information is non-redundant), signs with no mouth action, and signs with echo phonology. Yet the contrasts found suggest this is not the case.

**Figure 5:**
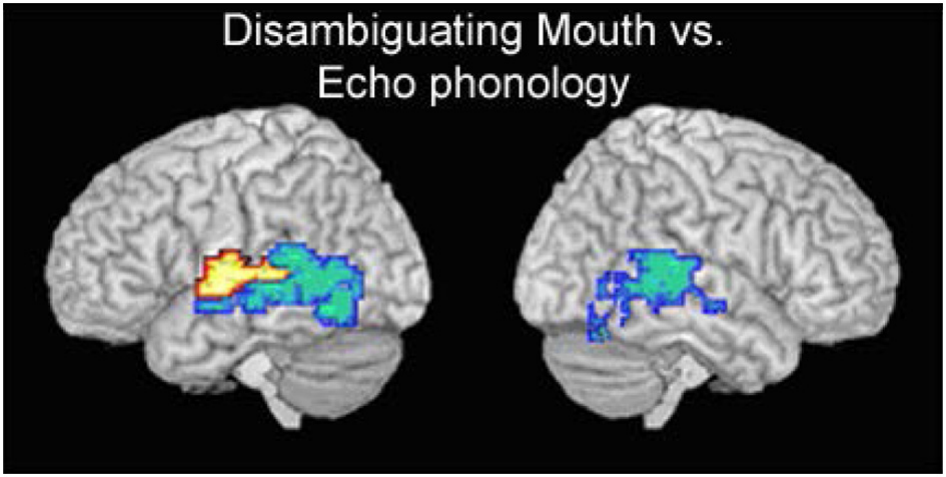
**Contrast between activation for DM (yellow) and EP signs (blue)**.

DM generated relatively greater activation in a circumscribed region of the left middle and posterior portions of the superior temporal gyrus (resembling the speech reading condition), while EP produced relatively greater posterior activation (Capek et al., 2008, p. 1231). We can consider the four conditions to represent a continuum from speech (SR) to speech accompanying signs (DM) to signs with accompanying non-speech-like mouth actions (EP) to purely manual signs (Man). Since greater posterior activation is characteristic of more sign-like material, EP also occupies an intermediate position between signs without mouth and signs with mouth actions derived from spoken language (Figure 5) in terms of neural activity.

The comparison of mouthings (DM) and echo phonology (EP) provides information about the nature of the mouth movements, and their role in sign language processing. The only differences in activation between DM and EP signs were found in the temporal lobe, with echo phonology (which is not derived from speech) demonstrating relatively greater posterior activation in both hemispheres than DM. This can be interpreted as a cortical correlate of the claim that the hands are indeed “the head of the mouth” (Boyes-Braem and Sutton-Spence, 2001), for echo phonology, as proposed by Woll (2001). While DM resembles speechreading in terms of functional cortical correlates, activation for EP resembles that for manual-only signs. Thus EP appears to occupy an intermediate position between spoken words and signs.

## Conclusions

One issue for those concerned with suggesting a link between gesture and word has always been how the arbitrary symbol-referent relationship of words in spoken language could have come from visually-motivated gestures. Echo phonology provides evidence for a possible mechanism. Firstly, the phenomenon appears to be fairly common across different sign languages (although the occurrence of echo phonology remains to be researched in non-European sign languages). Secondly, the mouth actions found in echo phonology are themselves non-visually motivated. For example, signers report that BSL exist is iconic (Vinson et al., 2008), indicating “something located there,” but it is impossible to reconstruct from the echo phonology syllable which accompanies it [⨜] the meaning “exist,” Thirdly, the actual inventory of elements in echo phonology looks very much like a system of maximal contrasts in a spoken language phonology (although there are some limitations because of the absence of sound contrasts). Fourthly, functional imaging research on the representation of signs and words in the brain suggests that echo phonology occupies an interesting intermediate position.

This paper represents a preliminary exploration of echo phonology. However, the data lead us to a number of conclusions. They support the arguments of those who argue against the notion that a unimodal manual protolanguage preceded the evolution of spoken language, since they demonstrate the extent to which signs are combined with mouth actions. The data also provide a window onto a mechanism by which the arbitrary pairing of a referent with a symbol (Saussure's defining feature of spoken language) could have occurred. Further research is needed to explore the presence of echo phonology in other sign languages (including those with a more recent point of creation than BSL) and whether echo phonology is subject to change (for example, added or transformed in a process of sign conventionalization). These studies may provide more insights into the origins of phonological/lexical structure in spoken language, and from that to the evolution of human language.

### Conflict of interest statement

The author declares that the research was conducted in the absence of any commercial or financial relationships that could be construed as a potential conflict of interest.

## Footnotes

^1^“Deaf” with an upper-case “D” is used to refer to membership of a sign language-using community and includes both hearing and (audiologically) deaf individuals.
